# Paradoxical Sensitivity to an Integrated Stress Response Blocking Mutation in Vanishing White Matter Cells

**DOI:** 10.1371/journal.pone.0166278

**Published:** 2016-11-03

**Authors:** Yusuke Sekine, Alisa Zyryanova, Ana Crespillo-Casado, Niko Amin-Wetzel, Heather P. Harding, David Ron

**Affiliations:** Cambridge Institute for Medical Research, University of Cambridge, Cambridge, United Kingdom; University of British Columbia, CANADA

## Abstract

The eukaryotic translation initiation factor eIF2B promotes mRNA translation as a guanine nucleotide exchange factor (GEF) for translation initiation factor 2 (eIF2). Endoplasmic reticulum (ER) stress-mediated activation of the kinase PERK and the resultant phosphorylation of eIF2’s alpha subunit (eIF2α) attenuates eIF2B GEF activity thereby inducing an integrated stress response (ISR) that defends against protein misfolding in the ER. Mutations in all five subunits of human eIF2B cause an inherited leukoencephalopathy with vanishing white matter (VWM), but the role of the ISR in its pathogenesis remains unclear. Using CRISPR-Cas9 genome editing we introduced the most severe known VWM mutation, *EIF2B4*^*A391D*^, into CHO cells. Compared to isogenic wildtype cells, GEF activity of cells with the VWM mutation was impaired and the mutant cells experienced modest enhancement of the ISR. However, despite their enhanced ISR, imposed by the intrinsic defect in eIF2B, disrupting the inhibitory effect of phosphorylated eIF2α on GEF by a contravening *EIF2S1*/eIF2α^*S51A*^ mutation that functions upstream of eIF2B, selectively enfeebled both *EIF2B4*^*A391D*^ and the related severe VWM *EIF2B4*^*R483W*^ cells. The basis for paradoxical dependence of cells with the VWM mutations on an intact eIF2α genotype remains unclear, as both translation rates and survival from stressors that normally activate the ISR were not reproducibly affected by the VWM mutations. Nonetheless, our findings support an additional layer of complexity in the development of VWM, beyond a hyperactive ISR.

## Introduction

Exchange of GDP for GTP on translation initiation factor 2 (eIF2) is an important determinant of rates of global protein synthesis in eukaryotes [1]. The activity of eIF2B, the guanine nucleotide exchange factor (GEF) charged with this task, is regulated in large part by the level of phosphorylation on the alpha subunit of its substrate (eIF2α). Phosphorylated eIF2α [eIF2(αP)] attenuates GEF activity, resulting in reduced global protein synthesis and decreased burden on the cell’s protein folding machinery [2, 3]. At the same time, eIF2(αP) and the attendant decrease in eIF2B GEF activity leads to enhanced translation of rare mRNAs that encode potent transcription factors that activate a gene expression program known as the Integrated Stress Response (ISR) in animals [4] or the General Control Response in yeast [5].

The circuitry involved in regulating the ISR, namely the kinases that phosphorylate eIF2α and the phosphatases that de-phosphorylate it, are carefully controlled. In both directions, altered function of the ISR affects fitness: abnormally low ISR activity exposes cells to the risk of protein misfolding due to unregulated protein synthesis whereas abnormally high ISR activity leads to a failure to maintain adequate levels of protein synthesis [2, 3, 6]. Balance in the ISR is especially important to fitness of the nervous system: pathological demyelination is exacerbated by abnormally low ISR activity [7] whereas cognitive dysfunction in certain neurodegenerative models is relieved by reduced ISR activity [8, 9].

The genes encoding the five subunits of the GEF eIF2B are essential. However in humans, diverse recessive missense mutations in all five are causally linked to the development of a leukoencephalopathy, known as Vanishing White Matter (VWM) or Childhood Ataxia with Cerebral Hypomyelination (CACH) [10, 11]. The recessive inheritance and the scattering of mutations widely throughout the coding sequences of the subunits are most in line with a loss-of-function phenotype [12]. This notion is supported by a trend for lower GEF activity in lysates from patient derived cells [13] and an enhanced ISR [14]; though the correlation between these biochemical features and disease severity is imperfect [15, 16].

Despite these clues, the role, if any, of altered ISR activity in the pathophysiology of VWM has remained elusive. Here we exploited the power of CRISPR-Cas9-induced recombination to create isogenic cell lines with and without a severe VWM mutation (*EIF2B4*^*A391D*^). Further genetic manipulation of the ISR revealed an unanticipated dependence of the VWM cells on signaling by phosphorylated eIF2α, despite their heightened ISR. We attempt to place this paradox within the context of cells that cope with fluctuating levels of unfolded protein stress.

## Results

### An isogenic cell culture model of a severe VWM-linked *EIF2B4^A391D^* mutation

**Fig 1:**
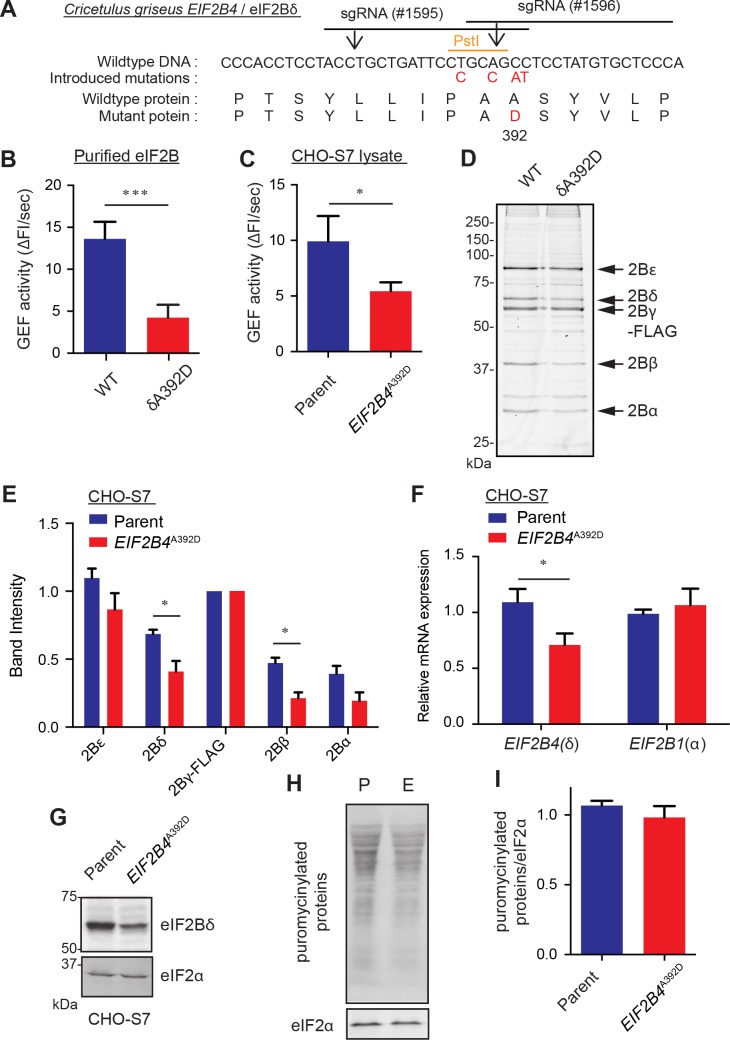
A cell model for the human VWM disease-linked mutation *EIF2B4*^*A391D*^. (A) The *Cricetulus griseus EIF2B4* genomic locus (NW_003613640.1, 3817:3861) targeted by CRISPR-Cas9 system and eIF2Bδ encoded protein (XP_003497100.1, 383:397). Horizontal lines and vertical arrows represent the sgRNA binding sites and Cas9 cleavage sites, respectively. Mutations, shown in red, disrupt sgRNA targeting, eliminate a *Pst*I site and generate an A392D (corresponding to human A391D) coding sequence mutation. (B) GEF activity of purified wildtype (WT) or eIF2Bδ^A392D^ mutant (δA392D) eIF2B complex. Shown are means ± S.D. of six independent experiments. *** P = 6.78 x 10^−6^, Unpaired t test. (C) GEF activity in the lysates of parental CHO-S7 and *EIF2B4*^*A392D*^ mutant cells. Shown are means ± S.D. of three independent experiments. * P = 0.032, Unpaired t test. (D) Coomassie brilliant blue stained SDS-PAGE of the endogenous eIF2B complex purified from parental CHO-S7 and *EIF2B4*^*A392D*^ cells by affinity chromatography of the FLAG-tagged endogenous eIF2Bγ subunit. The position of the individual subunits is indicated. (E) Quantification of the relative band intensity of eIF2B component in “C”, normalized to eIF2Bγ-FLAG signal. Shown are means ± SEM of three independent experiments. * P = 0.028 and 0.0103 for 2B4 and 2B2, respectively, Unpaired t test. (F) Quantitative PCR for mRNA expression of *EIF2B4* and *EIF2B1* in parental CHO-S7 and *EIF2B4*^*A392D*^ cells. Shown are means ± S.D. of three independent experiments. * P = 0.013, Unpaired t test. (G) Immunoblot of eIF2Bδ and eIF2α in parental CHO-S7 and *EIF2B4*^*A392D*^ cells. (H) Immunoblot of puromycinylated proteins following a brief pulse of puromycin, reporting on levels of translation. P and E indicate parental CHO-S7 and *EIF2B4*^*A392D*^ mutant cells, respectively. (I) Quantification of the puromycinylated proteins in “H”, normalized to an internal control, eIF2α. Shown are means ± SEM from three experiments.

Cell biological characterization of VWM mutations has relied upon patient derived cell lines. To circumvent the confounding effects of background genotypic and phenotypic variation inherent in such studies we used CRISPR-Cas9 gene editing to create isogenic wildtype Chinese hamster ovary (CHO) cells and cells bearing a severe VWM mutation corresponding to Alanine 391 to Aspartic acid on human eIF2Bδ subunit (*EIF2B4*^A392D^ in CHO) [10]. To facilitate detection of the mutant allele we introduced silent mutations that create a restriction fragment length polymorphism (RFLP), alongside the disease-associated missense mutation (Fig 1A). We generated two independent *EIF2B4*^A392D^ mutant cell clones derived from different CHO parental cells; UPR-reporter containing S21 cells and S7 cells with a FLAG-tagged endogenous eIF2Bγ subunit (see below).

The mutation had conspicuous hypomorphic features, imparting an intrinsic defect in GEF activity on the purified eIF2B complex (Fig 1B and S1A Fig). Lower levels of GEF activity were also noted in lysates of mutant cells (Fig 1C), which was consistent with the combined intrinsic defect in GEF activity noted above and mild instability of the mutant eIF2B complex (Fig 1D and 1E and S1B Fig). The latter correlated with lower levels of *EIF2B4* mRNA and protein in the mutant cells (Fig 1F and 1G). These were observed in independently derived mutant clones elaborated in different CHO parental lines (S1C–S1E Fig), but affected neither baseline levels of global protein synthesis (Fig 1H and 1I and S1F Fig) nor cell proliferation (S1G Fig).

**Fig 2:**
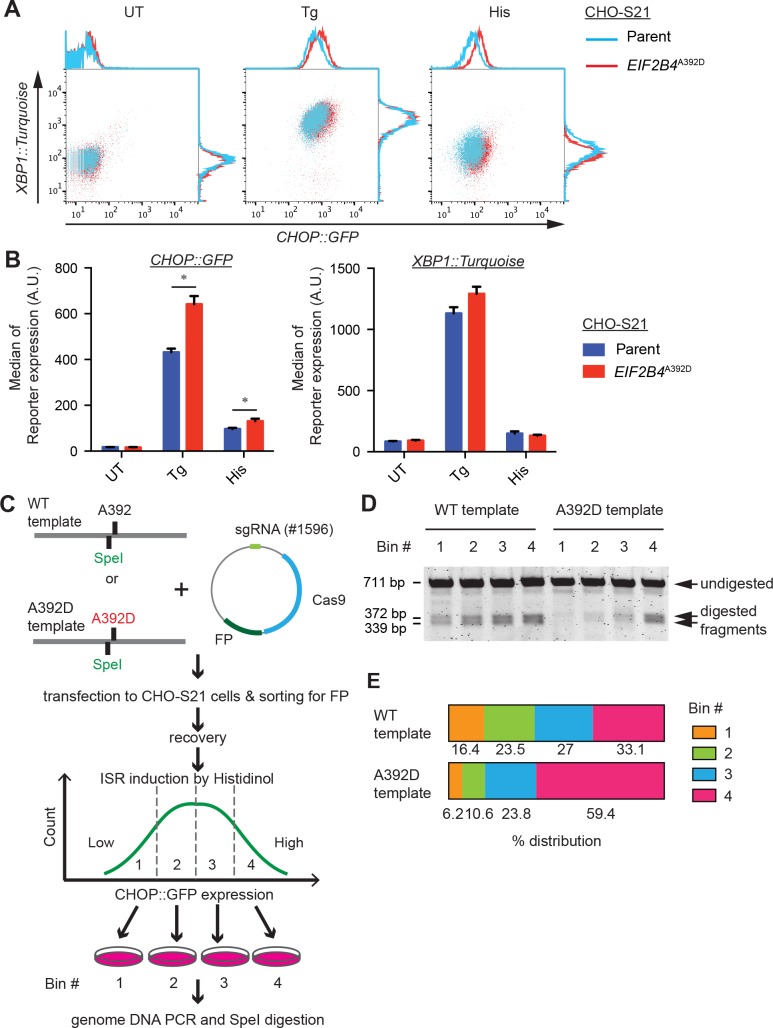
Heightened ISR in *EIF2B4*^*A392D*^ cells. (A) Flow cytometry analysis of *CHOP*::*GFP* and *XBP1*::*Turquoise* dual reporter-containing parental CHO-S21 and *EIF2B4*^*A392D*^ mutant cells. The cells were untreated (UT) or stimulated with 250 nM thapsigargin (Tg) or 0.5 mM histidinol (His) for 24 hours. Note the enhanced response of the *CHOP*::*GFP* ISR reporter. (B) Bar diagram of the median ± S.D. of the reporter gene activity from experiments as shown in “A”. N = 3, *P = 0.0057 for Tg, *P = 0.037 for His, Unpaired t test. (C) Experimental design for tracking *EIF2B4*^*A392D*^ mutations. A fluorescent protein-marked sgRNA/Cas9 plasmid targeting *EIF2B4* and a wildtype or *EIF2B4*^*A392D*^ mutant repair template marked by a silent *Spe*I mutation were co-transfected into CHO-S21 cells. Transfected cells (selected by FACS), were treated with histidinol and divided into four bins (Bin #1 to #4) by level of *CHOP*::*GFP* expression. After recovery, genomic DNA was isolated from cells in each bin and the targeted region of *EIF2B4* was amplified by PCR and digested with *Spe*I to reveal frequency of targeting by either repair template. (D) PCR fragments digested with *Spe*I from genomic DNA of the indicated bins, visualized on an agarose gel. Shown is an image of a representative experiment reproduced twice. (E) Plot of the distribution of *Spe*I digested fragments in the four bins of transduced cells from the experiment in “D”. The band intensities of the digested fragments (reporting on recombination of the wildtype or mutant repair template) were normalized to total PCR product intensity and the distribution of the relative frequency of recombination in the different bins was plotted. Note the enrichment for recombination of the *EIF2B4*^*A392D*^ mutant repair template in the ISR^High^ bin.

To gauge the effect of the *EIF2B4*^*A392D*^ mutation on the activity of the ISR, we made use of a resident *CHOP*::*GFP* ISR reporter transgene [17]. The basal activity of *CHOP*::*GFP* was indistinguishable in wildtype and *EIF2B4*^*A392D*^ mutant cells. However induction of endoplasmic reticulum (ER) stress by the ER calcium depleting agent thapsigargin (that activates the eIF2α kinase PERK) or by histidinol, an inhibitor of histidyl tRNA synthetase (that activates the eIF2α kinase GCN2), led to conspicuously more signaling in the ISR of the mutant cells (Fig 2A and 2B). Enhanced signaling in the mutant cells was specific for the ISR. Thapsigargin-mediated activity of an *XBP1*::*Turquoise* transgene (that reports on a parallel IRE1-dependent branch of the ER stress response [18]), was unaffected by the mutation. In histidinol-treated cells the induction of *XBP1*::*Turquoise* was slightly but reproducibly attenuated by the mutation, likely a reflection of feed-back emanating from the enhanced ISR activity (Fig 2A and 2B).

To minimize the effect of clonal selection on the heightened ISR activity observed in *EIF2B4*^*A392D*^ cells, we devised an unbiased assay to measure the ISR in polyclonal populations of cells that had been induced to acquire the *EIF2B4*^*A392D*^ mutation. Parental S21 cells were co-transfected with fluorescent-tagged Cas9 and a guide targeting the *EIF2B4* locus, alongside either wildtype or *EIF2B4*^*A392D*^ repair templates that harbor an *Spe*I RFLP distinguishing a recombinant *EIF2B4* locus from a parental one (Fig 2C and S2A Fig). The frequency of homologous recombination by this transient transfection method was low and the pool of cells offered the mutant repair template had only a minimally-elevated ISR compared to those offered a wildtype repair template (S2B Fig). The transiently transfected cells were then divided by FACS into four bins covering the range of ISR activity and the frequency of homologous recombination with either the wildtype or *EIF2B4*^*A392D*^ repair template was measured in each bin (Fig 2C). Cells that had recombined the *EIF2B4*^*A392D*^ repair template were enriched in the ISR^High^ bin (#4), whilst the frequency of homologous recombination with the wildtype repair template was more evenly distributed amongst the four bins (Fig 2D and 2E). These observations further reveal that the *EIF2B4*^*A392D*^ mutation disposes cells to an enhanced ISR.

### Severe VWM mutant cells are intolerant of an ISR-enfeebling *EIF2S1*^*S51A*^ mutation

Mutations in eIF2B that enhance the yeast counterpart of the ISR (the General Control Response) buffer the effect of other mutations that lower levels of eIF2(αP) [19]. To test for similar buffering in VWM mutant cells, we sought to compare the phenotypic consequences of interfering with eIF2(αP) in wildtype and two severe VWM models: the aforementioned *EIF2B4*^*A392D*^ and *EIF2B4*^*R484W*^, which corresponds to the human VWM mutation R483W [10, 11, 12] (S3A and S3B Fig).

**Fig 3:**
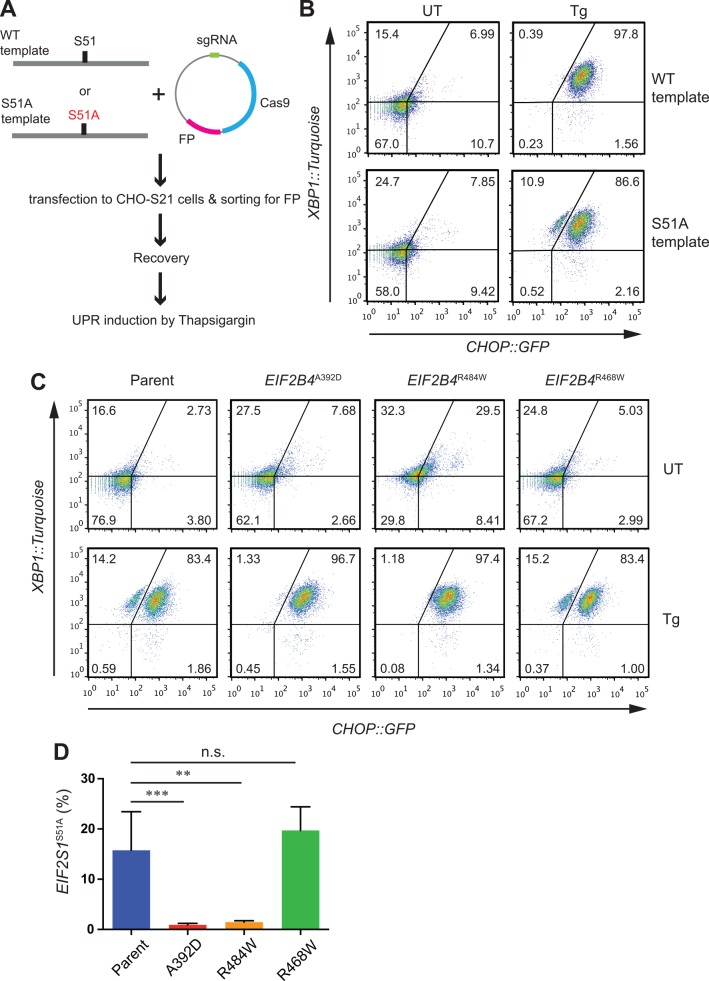
Severe VWM mutant cells are unable to tolerate a second *EIF2S1*^*S51A*^ mutation. (A) Experimental design for tracking *EIF2S1*^*S51A*^ mutant cells. A fluorescent-tagged sgRNA/Cas9 plasmid targeting *EIF2S1* was co-transfected alongside wild type (WT) or *EIF2S1*^*S51A*^ (Mut) templates into CHO-S21 dual reporter cells. Following FACS selection for the transfected cells they were treated with 250 nM thapsigargin (Tg) for 24 hours and reporter expression was analyzed. (B) Flow cytometry analysis of reporter activity in untreated (UT) and thapsigargin-treated (Tg) CHO-S21 cells from the experiment outlined in “A”. Note the emergence of *CHOP*::*GFP* negative, *XBP1*::*turquoise* positive thapsigargin-treated cells in the pool offered an *EIF2S1*^*S51A*^ repair template. (C) Flow cytometry analysis of reporter activity in untreated (UT) and thapsigargin-treated (Tg) parental CHO-S21 or indicated VWM mutant cells following targeting of the *EIF2S1* locus with an *EIF2S1*^*S51A*^ repair template (as described in “A”). Note the lack of *CHOP*::*GFP* negative, *XBP1*::*turquoise* positive thapsigargin-treated putative *EIF2S1*^*S51A*^*; EIF2B4*^*A392D*^ or *EIF2S1*^*S51A*^*; EIF2B4*^*R484W*^ double mutant cells (lower right panel). (D) Percentage of *CHOP*::*GFP* negative, *XBP1*::*turquoise* positive thapsigargin-treated putative *EIF2S1*^*S51A*^ mutant cells in the indicated population from experiments as in “C”. Shown are means ± S.D. N = 6 (Parent), 5 (*EIF2B4*^*A392D*^), and 3 (*EIF2B4*^*R484W*^ and *EIF2B4*^*R468W*^). *** P<0.001, ** P<0.01, n.s. not significant, One way ANOVA followed by Dunnett’s multiple comparisons test.

CRISPR-Cas9 guides targeting the *EIF2S1/*eIF2α locus were transfected alongside a repair template with a Ser 51 to Ala mutation in eIF2α that blocks signaling to the ISR by upstream stress-activated kinases [20] (Fig 3A). In CHO cells wildtype at the *EIF2B4* locus, this led to the emergence of a subpopulation of cells with impaired induction of the *CHOP*::*GFP* ISR reporter upon exposure to the ER stress-inducing agent thapsigargin. Though not studied in further detail here, the defect in the ISR likely arises both in cells that have acquired homozygous *EIF2S1*^*S51A*^ mutation and in cells in which the *EIF2S1*^*S51A*^ allele is present in trans to a null. Heterozygous cells with a wildtype allele of *EIF2S1* are likely to be phenotypically wildtype [20]. The defect imposed by *EIF2S1*^*S51A*^ mutation was selective for the ISR, as the parallel stress-induced pathway mediated by IRE1α and reported on by the *XBP1*::*Turquoise* reporter was unaffected (Fig 3B).

*EIF2S1*^*S51A*^ mutant cells are hypersensitive to stress. This was reflected in the depletion of the ISR negative (*EIF2S1*^*S51A*^ mutant) pool from a mixed population of wildtype and mutant cells, following exposure to histidinol (S3C and S3D Fig). Nonetheless *EIF2S1*^*S51A*^ mutant cells gave rise to clones that could be propagated indefinitely (S3E Fig). By contrast *EIF2B4*^*A392D*^ mutant cells targeted with the same *EIF2S1*^*S51A*^ repair template failed to elaborate a sizeable pool of *CHOP*::*GFP* uninduced cells (Fig 3C and 3D & S3F and S3G Fig) and the few *CHOP*::*GFP* dim cells observed transiently after transduction of the *EIF2S1*^*S51A*^ repair template failed to give rise to *EIF2B4*^*A392D*^; *EIF2S1*^*S51A*^ double mutant clones. *EIF2B4*^*R484W*^ mutant cells were similarly unable to acquire the *EIF2S1*^*S51A*^ mutation (Fig 3C and 3D). By contrast CHO cells with a milder VWM mutation, *EIF2B4*^*R468W*^ (a model of human *EIF2B4*^*R467W*^ [21], S3A and S3B Fig) had no difficulty accommodating a second *EIF2S1*^*S51A*^ mutation (Fig 3C and 3D). This feature of severe VWM mutant cells likely reflected a specific inability to tolerate the *EIF2S1*^*S51A*^ mutation, as opposed to altered susceptibility to CRISPR-Cas9 gene editing, as targeting the *ERN1*/IRE1α locus with CRISPR-Cas9 guides gave rise to a similar population of *XBP1*::*Turquoise* negative daughters in wildtype or *EIF2B4*^*A392D*^ parents (S3H and S3I Fig). Furthermore, as this analysis is conducted on pools of cells with no selection (beyond that for transient expression of the CRISPR-Cas9 encoding plasmid), the phenotypic difference between the wildtype and severe VWM mutation-bearing cells are unlikely to have arisen from random clonal selection.

**Fig 4:**
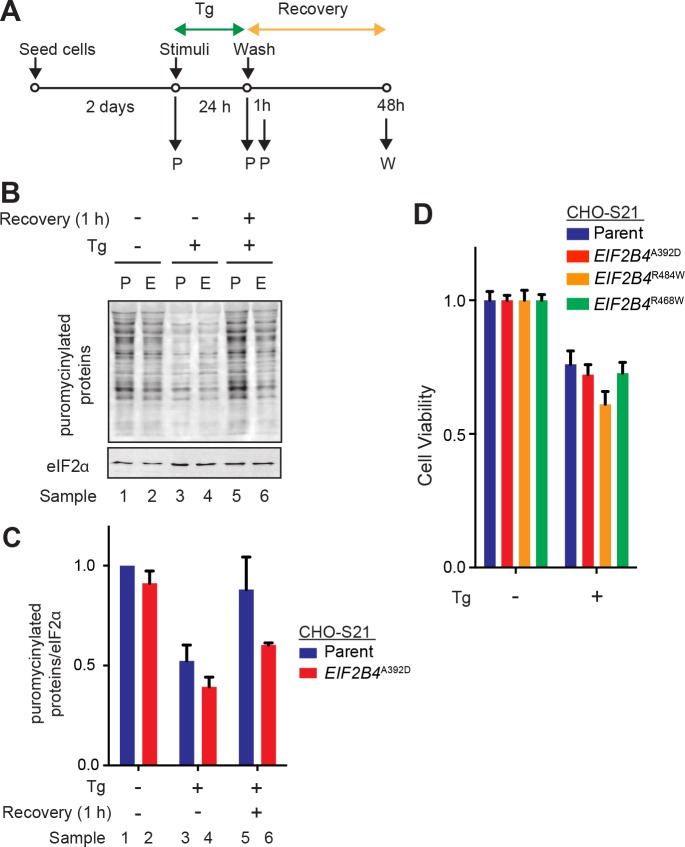
Stress-resistance of wildtype and VWM cells. (A) Schema of experiments to compare the effect of thapsigargin in parental CHO-S21 and VWM mutant cells. Cells were treated with thapsigargin (Tg; 250 nM) for the indicated time, washed free of compounds and allowed to recover before assay. W = WST-1 assay, P = Puromycin labeling. (B) Immunoblot of puromycinylated proteins following a brief pulse of puromycin, reporting on levels of translation under the indicated experimental conditions. Shown is a representative experiment reproduced four times. P and E indicate parental CHO-S21 and *EIF2B4*^*A392D*^ mutant cells, respectively. (C) Quantification of “B”. Signal intensities of puromycinylated proteins were normalized by eIF2α. Shown are means ± SEM of four independent experiments. (D) Cell viability measured by the WST-1 assay in the experiment described in “A”. Shown are the mean ± SEM of four replicates of a representative experiment repeated three (R484W, R468W) to six (A392D) times.

Despite the conspicuous dependence of severe VWM mutant cells on an intact ISR, we observed no consistent difference in the magnitude of the repression of protein synthesis by thapsigargin between wildtype and mutant cells (Fig 4A–4C). A modest defect in the recovery of protein synthesis in the mutant cells following washout of the thapsigargin in some experiments (Fig 4B, lanes 5 & 6), was not consistently observed in all experiments. Furthermore, the survival of cells following exposure to thapsigargin, was unaffected by the VWM mutations (Fig 4D), leaving no simple explanation for the strong genetic interaction between the VWM mutation and the *EIF2S1*^*S51A*^ genotype.

## Discussion

Stress, imposed by infection or head trauma is frequently cited as a precipitating event in VWM [22]. But hypersensitivity to stress has not been evident in cell culture models of the disease. Both theoretical considerations (built on the hypomorphic features of disease-associated mutations in eIF2B) and empirical observations suggest, if anything, that VWM cells have heightened activity of the ISR–a major stress resistance pathway that counteracts proteotoxicity. To help address this conundrum and detect potentially subtle disease-associated phenotypes we chose to introduce severe, early onset VWM mutations into otherwise isogenic CHO cell lines with built-in reporters for the UPR and ISR.

Whilst CHO cells are a limited model for the complex neurobiology of VWM, they do provide a useful tool to elucidate general aspects of the effect of the mutation. Further important advantages of CHO cells are their relative clonal stability, the availability of integrated ISR reporters and the ease of CRISPR-Cas9 gene editing in these cells (this is an important improvement over lymphoblast cell lines isolated from patients used in previous studies of VWM, as the latter lack isogenic wildtype counterparts and are subject to clonal variation during immortalization). Finally, severe VWM mutations also compromise organs such as the female ovary [23] and CHO cells, derived from hamster ovaries, might capture some of the relevant biology. Thus, whilst one may not safely extrapolate from CHO cells to the astrocytes and oligodendrocytes that are the target cells implicated in VWM, it seems reasonable to imagine that the fundamental biology is shared.

In an isogenic context, where gene dosage affects have been eliminated, we confirmed that a severe VWM model in CHO cells is associated with lowered eIF2B GEF activity. The basis for this is likely multifactorial as the mutation studied in detail, *EIF2B4*^*A392D*^, negatively affected both the abundance of the eIF2B complex and its intrinsic GEF activity. This feature likely accounts for the enhanced ISR activity of the VWM cells; a subtle phenotype, but one that is observed both in single mutant clones (where clonal variation is always a concern) and in unselected pools of mutant cells.

The inability of cells with either of two severe VWM mutations (*EIF2B4*^*A392D*^ and *EIF2B4*^*R484W*^) to tolerate a second mutation in eIF2α that prevents its phosphorylation on serine 51 and thus eliminates ISR activity (*EIF2S1*^*S51A*^), occurred against this backdrop of diminished eIF2B activity and a heightened ISR activity of the VWM cells. This is a robust phenotype, observed in multiple *EIF2B4*^*A392D*^ clones and in the related severe *EIF2B4*^*R484W*^ mutation. By contrast CHO cells with a weaker *EIF2B4*^*R468W*^ VWM mutation, readily tolerated the ISR-blocking *EIF2S1*^*S51A*^ mutation, suggesting a correlation between the severity of the VWM mutation and the dependence of the cells on a serine residue in position 51 of eIF2α.

The mechanism by which the severe VWM mutations sensitize cells to the loss of ISR signaling through an *EIF2S1*^*S51A*^ mutation remains unclear. It is formally possible that an alanine in position 51 of eIF2α has hypomorphic features that are unrelated to the loss of the ISR-initiating phosphorylation event at that site; features that become critical in presence of a second, severe VWM mutation. However, in mice, the *EIF2S1*^*S51A*^ mutation is largely mimicked by mutations in the upstream kinases [20] leading us to disfavor this possibility. An alternative explanation might be that severe VWM mutations are impaired in tolerating severe swings in unfolded protein stress that occur when the ISR is dysregulated, but this possibility will need to be explored in further detail.

## Materials and Methods

### Cell culture and Reagents

CHO-K1 (ATCC, CCL-61) based cell lines were maintained in regular media consisting of Nutrient Mixture F12 Ham (SIGMA), 10% Fetal calf serum (FetalClone II, ThermoFisher), 2 mM L-glutamine (SIGMA), and 1x Penicillin/Streptomycin at 37°C with 5% CO_2_.

CHO-S21 cells containing the *CHOP*::*GFP* and *XBP1*::*Turquoise* reporters were derived from G418^r^ *CHOP*::*GFP* CHO-C30 cells [17] by introduction of a stable *XBP1*::*Turquoise* reporter derived by replacing the Venus fluorescent protein in pCAX-F-XBP1ΔDBD-venus [18] with Turquoise alongside a puromycin resistance marker and selection for clones that activate both reporters in response to ER stress.

Reagents were sourced as indicated: thapsigargin (Calbiochem), L-histidinol dihydrochloride (ACROS Organics), puromycin (Calbiochem), and tunicamycin (MELFORD).

### Generation of the genome edited cells by CRISPR-Cas9

#### CHO-S7 cells

A 3xFLAG tag was incorporated into the *EIF2B3*/eIF2Bγ subunit of CHO-C30 cells [17] by a CRISPR-Cas9 mediated homology-directed repair (HDR). A single guide (sg) RNA sequence for targeting C-terminal CDS of *EIF2B3* was selected from the CRISPy database [URL: http://staff.biosustain.dtu.dk/laeb/crispy/ [24]] and a duplex DNA of the sequence (made of oligo DNA No. 1 and No. 2 in S1 Table) was inserted into the pCas9(BB)-2A-GFP plasmid (Addgene plasmid #48138) following published procedures [25], making the sgRNA/Cas9 plasmid (lab ID; UK1491). To construct a repair template containing the 3xFLAG sequence at the C-terminal end of *EIF2B3* CDS without a stop codon, 5’ and 3’ homology arms were amplified from CHO genomic DNA by PCR using the primer sets; oligo DNA No. 3 & 4 and oligo DNA No. 5 & 6, respectively. A 3xFLAG sequence was recovered from the pCEFL_mCherry_3xFLAG_C plasmid (lab ID; UK1314) digested with EcoRI and ApaI. The 5’ homology arm (digested with SacI/EcoRI), 3’ homology arm (digested with ApaI/KpnI), and 3xFLAG sequence were sequentially ligated into a correspondingly digested pBS KS(+) plasmid (lab ID; UK1), making a repair template plasmid (lab ID; UK1500).

CHO-C30 cells were plated in 6 well plates at a density of 2 x 10^5^ per well. 24 hours later the cells were transfected with the repair template plasmid (1 μg) and an sgRNA/Cas9 plasmid (1 μg) using Lipofectamine LTX (Invitrogen) according to manufacturer’s instruction. The next day, GFP positive cells, which indicate plasmid transfected cells, were collected using a Beckman Coulter MoFlo Cell sorter, and expanded as single clones in 96 well plates. Genomic DNAs isolated from the clones were screened by PCR using primers (oligo DNA No. 7 & 8) and EcoRI digestion (3xFLAG inserted cell should have exogenous EcoRI site at C-terminus of *EIF2B3* CDS derived from the repair template). Finally, the insertion of 3xFLAG was confirmed by sequencing. The clone (S7) we used in this study has an insertion of 3xFLAG in one allele while another allele has intact *EIF2B3*.

#### CHO *EIF2B4*^*A392D*^, *EIF2B4*^*R484W*^ and *EIF2B4*^*R468W*^ cells

The *EIF2B4*^A392D^ mutation was incorporated into the CHO-S21 cells (see Cell culture section) or CHO-S7 (see above) cells by a similar CRISPR-Cas9 mediated HDR as above. The duplex DNAs [made of oligo DNAs (No. 9 and No. 10) or (No. 11 and No. 12) in S1 Table] were inserted into the pCas9(BB)-2A-GFP plasmid, making the sgRNA/Cas9 plasmids (lab ID; UK1595, or UK1596, respectively). For a repair template, 5’ and 3’ homology arms amplified by oligo DNAs [(No. 13 and No. 14) and (No. 15 and No. 16), respectively] were PCR knitted by primers No. 13 and No. 16. PCR products were purified from agarose gel using GeneJET Gel Extraction Kit (ThermoFisher) and used as a repair template. Transfection, cell sorting and cell cloning were similarly done as described above. For genotyping screen at *EIF2B4* A392 locus, genomic DNA was amplified by nested PCR using oligo DNAs No. 17 and No. 18 as a first primer set and No. 13 and No. 16 as second. The PCR products were digested by *Pst*I because the repair template contains silent mutations to disrupt the endogenous *Pst*I site for a marker of HDR as shown in Fig 1A. The incorporated mutations were confirmed by sequencing for genomic DNA and cDNA derived from mutant clones. We obtained clones harboring homozygous *EIF2B4*^*A392D*^ mutation in CHO-S21 cells (c6) from the UK1595 transfected clones, and CHO-S7 cell line (c3) from the UK1596 transfected clones. The *EIF2B4*^*R484W*^ or *EIF2B4*^*R468W*^ mutation in CHO-S21 cells was generated using similar methods as *EIF2B4*^A392D^. For *EIF2B4*^*R484W*^, the duplex DNAs [made of oligo DNAs (No. 33 and No. 34) in S1 Table] were inserted into the pCas9(BB)-2A-mCherry plasmid, making the sgRNA/Cas9 plasmids (lab ID; UK1633). For a repair template, 5’ and 3’ homology arms amplified by oligo DNAs [(No. 35 and No. 36) and (No. 37 and No. 38), respectively] were PCR knitted by primers No. 35 and No. 38. For *EIF2B4*^*R468W*^, the duplex DNAs [made of oligo DNAs (No. 39 and No. 40) in S1 Table] were inserted into the pCas9(BB)-2A-GFP plasmid, making the sgRNA/Cas9 plasmids (lab ID; UK1597). For a repair template, 5’ and 3’ homology arms amplified by oligo DNAs [(No. 35 and No. 41) and (No. 42 and No. 38), respectively] were PCR knitted by primers No. 35 and No. 38.

#### *EIF2B4*^*A392D*^ mutation pool analysis in CHO-S21 cells

For the *EIF2B4*^A392D^ mutation repair template, 5’ and 3’ homology arms amplified by oligo DNAs [(No. 13 & No. 31) and (No. 15 & No. 16), respectively] were PCR knitted by primers No. 13 and No. 16. For A392 wildtype control, oligo DNA No. 32 was used instead of No. 31. PCR products were purified using GeneJET Gel Extraction Kit (ThermoFisher) and used as a repair template.

CHO-S21 cells were plated in 6 well plates at a density of 2 x 10^5^ per well. 24 hours later the cells were transfected with the repair template plasmid (0.5 μg) and an sgRNA/Cas9 plasmid (UK1596, 0.5 μg) using Lipofectamine LTX (Invitrogen). Two days later, GFP positive cells, which indicate plasmid transfected cells, were collected by using a Beckman Coulter MoFlo Cell sorter, and recovered in the medium for five to six days till the fluorescent marker expression was eliminated. The cells were re-plated in 6 well plates at a density of 4 x 10^4^ cells per well and two days later, the cells were treated with 0.5 mM histidinol. After 18 hours, the cells were subjected to cell sorting using a Beckman Coulter MoFlo Cell sorter. Sorting gates were set according *CHOP*::*GFP* reporter expression as bin#1; top 10%, bin#2; upper middle 35%, bin#3 lower middle 35%, and bin#4 bottom 10% GFP expression, respectively (See also Fig 2C). Cells located between the gate boundaries (10% of total) were not collected. Collected cells were recovered and expanded to obtain enough cells for genomic DNA isolation.

Genomic DNA was isolated using standard phenol-chloroform extraction and isopropanol precipitation protocol. *EIF2B4* A392 genomic locus was amplified by nested PCR using oligo DNAs No. 17 and No. 18 as a first primer set and No. 13 and No. 16 as second. The PCR products were digested by *Spe*I (*Bcu*I, ThermoFisher) and run on 2% agarose gel containing SYTO60 red fluorescent nucleic acid stain (ThermoFisher). The gel was scanned on an Odyssey near infrared imager (LI-COR).

#### *EIF2S1*/eIF2α S51A mutation or *ERN1*/IRE1α deletion pool analysis in CHO-S21 cells

The duplex DNA for targeting *EIF2S1* S51A [made of oligo DNAs (No. 25 and No. 26) in S1 Table] or *ERN1* [made of oligo DNAs (No. 27 and No. 28)] were inserted into the pCas9-2A-mCherry plasmid [lab ID; UK1610, which was made by replacing the eGFP portion of pCas9(BB)-2A-GFP plasmid to mCherry using the Gibson assembly (NEB) according the manufacturer’s instruction], making the sgRNA/Cas9 plasmids (lab ID; UK1696, or UK1615, respectively). For a repair template of *EIF2S1* S51A or control S51 wildtype, single strand oligo DNAs (ssODN; No. 29 or No. 30 in S1 Table, respectively) were synthesized (Integrated DNA Technologies).

CHO-S21 cells were plated in 6 well plates at a density of 2 x 10^5^ per well. 24 hours later the cells were transfected with UK1696 plasmid (0.5 μg) and an ssODN (0.5 μg) for *EIF2S1* or UK1615 plasmid (0.5 μg) alone for *ERN1* using Lipofectamine LTX (Invitrogen). Two days later, mCherry positive cells, which indicate plasmid-transfected cells, were collected using a Beckman Coulter MoFlo Cell sorter, and recovered in the medium for five to six days. The cells were re-plated in 6 well plates at a density of 4 x 10^4^ cells per well, and two days later, the cells were treated with 250 nM thapsigargin for 24 hours. The reporter fluorescent protein expression in the cells was analyzed by Flow cytometry as described below.

### Quantitative PCR

Total RNA was isolated from CHO derived cells by acid guanidinium thiocyanate-phenol-chloroform extraction using RNA STAT 60 (Amsbio) and isopropanol precipitation. 1.5 μg of RNA was reverse transcribed by a reverse transcriptase RevertAid (ThermoFisher) with oligo(dT)18 primer. Quantitative PCR analysis was performed using Power SYBR Green PCR Master Mix (Applied Biosystems) according the manufacturer’s instruction on a 7900HT Fast Real-Time PCR system (Applied Biosystems). Oligo DNAs in Tabel I were used for PCR reaction; No. 19 and No. 20 for *EIF2B4*, No. 21 and No. 22 for *EIF2B1*, No. 23 and No. 24 for *RPL27*. Relative quantities of amplified PCR products were determined using SDS 2.4.1 software (Applied Biosystems) and normalized to *RPL27* values.

### Puromycin labeling and immunoblot analysis

For puromycin labeling experiments, 3 x 10^5^ cells were plated in 60 mm dishes. Two days later, culture media was changed to fresh media. For monitoring basal translation, the cells were treated with 10 μg/ml puromycin for 10 min and harvested. For monitoring translation in stimulated cells, cells were treated with 250 nM thapsigargin for 24 hours and 10 μg/ml puromycin was added during the last 10 min before harvest. For monitoring translation in recovering cells, the stimulated cells were washed by 3 ml of culture media and incubated for 1 hour, followed by treatment of 10 μg/ml puromycin during the last 10 min before harvest. Cells were lysed in a lysis buffer containing 1% Triton X-100, 50 mM Tris-HCl (pH 7.4), 150 mM NaCl, 1 mM EDTA, 10% Glycerol, 2 mM PMSF, 10 μg/ml aprotinin, 4 μg/μl pepstatin and 4 μM leupeptin. After centrifugation at 21130 x *g* for 15 min, supernatants were mixed with a standard SDS-PAGE sample buffer. 40 μg of total protein was subjected to 12.5% SDS–polyacrylamide gels electrophoresis and transferred onto Immobilin-P PVDF membrane (EMD Millipore). Immunoblot detection was conducted using primary antibodies for puromycinylated protein [26], total eIF2α [27], and IRDye 800 conjugated secondary antisera (LI-COR) followed by scanning on a Odyssey near infrared imager (LI-COR). Scanned images were quantified using imageJ software.

For detection of eIF2Bδ, a rabbit anti-eIF2Bδ primary antibody (Proteintech), HRP-linked rabbit secondary antibody and SuperSignal West Pico Chemiluminescent Substrate (ThermoFisher) were used.

### In vitro measurement of GEF activity on GDP-bound eIF2

The fluorescent intensity-based assay of GDP release from eIF2 (GEF assay) was performed as previously described [28]. Substrate eIF2 was prepared from 10 confluent 10 cm dishes of CHO cells harboring eIF2α^S51A^-3xFLAG. Cells were washed with ice cold PBS and collected with PBS containing 1 mM EDTA. After centrifugation at 376 x *g* for 5 min, the cell pellets were lysed with a 4 x volume of the lysis buffer described in the immunoblot analysis section. The lysates were centrifuged at 21130 x *g* for 15 min. The supernatant was pre-cleared with Protein A sepharose (ZYMED) at 4°C for 30 min. After removal of Protein A sepharose, 30 μl of FLAG-M2 sepharose (SIGMA) was added into the supernatant and rotated for 30 min at 4°C. The beads were washed with 1 ml of high salt (500 mM NaCl) lysis buffer three times and with 50 μl of GDP-mounting buffer [50 mM Tris-HCl pH7.4, 150 mM NaCl, 1 mM DTT] twice.

3xFLAG-eIF2 complex was eluted with 125 μg/ml 3xFLAG peptide (SIGMA) in 40 μl of GDP-mounting buffer at 4°C for 30 min mixing at 1000 rpm. The 40 μl of eluate, which contains approx. 30 nM eIF2 complex, was mixed with 20 μl of 150 nM Bodipy-FL-GDP (Invitrogen) and incubated at 25°C for 20 min to reach equilibrium binding of Bodipy-FL-GDP in eIF2. Then, the reactant was mixed with 10 μl of 12 mM MgCl_2_ and was passed through a G-50 sephadex column (GE healthcare) equilibrated with GEF assay buffer [50 mM Tris-HCl (pH7.4), 150 mM NaCl, 1 mM DTT, 2 mM MgCl_2_, 0.01% Triton X-100] and used as a substrate in the GEF assay.

CHO cells from 80–90% confluent 10 cm dishes were collected as described above and mixed with homogenization buffer [50 mM Tris-HCl (pH7.4), 375 μM magnesium acetate, 75 μM EDTA, 95 mM potassium acetate, 2.5 mg/ml digitonin, 10% Glycerol, 1 mM DTT] supplemented with protease and phosphatase inhibitors as the lysis buffer. The mixture was set on ice for 15 min and passed through a 0.5 ml syringe with 29G needle five times. After centrifugation at 21130 x *g* for 15 min, the supernatants were subjected to the GEF assay.

For the GEF assay, 2 μl of eIF2 substrate and 3 μl of GEF assay buffer containing 1.5 mM non-labeled GDP were mixed with 5 μl of 4.5 μg/μl cell lysate in 384 well round bottom black polystyrene assay plates (Corning, Cat #3667). Fluorescence intensity (excitation wavelength: 485 nm, bandwidth 20 nm, emission wavelength: 535 nm, band width 25 nm) was measured using a TECAN F500 plate reader every 20 seconds till the fluorescence intensity plateaued. GEF activity was calculated as a decrease of the fluorescent intensity (ΔFI) per second at the initial linear phase of the reaction.

### Immuno-affinity Purification of endogenous eIF2B complex

Fourteen 10 cm dishes of confluent CHO-S7 parental or *EIF2B4*^A392D^ cells were washed with ice cold PBS and collected with PBS containing 1 mM EDTA. After centrifugation at 376 x *g* for 5 min, the cell pellets were lysed with a 4 x volume of the modified homogenization (MH) buffer [50 mM Tris-HCl (pH7.4), 375 μM magnesium acetate, 75 μM EDTA, 95 mM potassium acetate, 10% Glycerol, 0.5% Triton X-100, 1 mM DTT] supplemented with protease and phosphatase inhibitors as the lysis buffer. The lysates were centrifuged at 21130 x *g* for 15 min. The supernatant was pre-cleared with Protein A sepharose at 4°C for 30 min. After removal of protein A sepharose (ZYMED), 30 μl of FLAG-M2 sepharose (SIGMA) was added into the supernatant and rotated for 30 min at 4°C. The beads were washed in 1 ml of MH buffer three times. FLAG-eIF2B complex on the beads was eluted with 250 μg/ml 3xFLAG peptide (SIGMA) in 20 μl of MH buffer at 4°C for 30 min mixing at 1200 rpm. After spinning down the beads, 18 μl of the supernatant was mixed with 6 μl of 4x SDS-sample buffer. The whole sample was subjected to 12.5% SDS-PAGE and the gel was stained by InstantBlue (Expedeon). The identity of eIF2B complex was confirmed by Mass spectrometry.

For a large scale purification of eIF2B complex, which was subjected to in vitro GEF assay as shown in Fig 1A and S1A and S1B Fig, CHO-S7 parental or *EIF2B4*^A392D^ cells were adapted to suspension culture. 3.5 litter of cultured cells (1 x10^6^ cells/mL) were collected by spin down and washed with 2 x 25 ml of ice cold PBS. Cells were lysed with 2x pellet volume of lysis buffer [50mM Tris pH7.5, 150mM NaCl, 5mM MgCl_2_, 0.5% Triton, 10% Glycerol, 1mM DTT, 1x protease inhibitors]. Lysate were clarified by centrifuge at 20,000 rpm for 30 min and ~ 35 ml of supernatant were obtained. 600 μl (300 μl bed volume) of FLAG-M2 sepharose (SIGMA) was added into the supernatant and rotated for 60 min at 4°C. The beads were washed with high salt (500 mM NaCl) lysis buffer four times and with GEF assay buffer four times. FLAG-eIF2B complex on the beads was eluted with 125 μg/ml 3xFLAG peptide (SIGMA) in 400 μl of GEF assay buffer twice. The eluted wildtype and mutant eIF2B complexes, which were adjusted to equal amount of eIF2Bε catalytic subunit (3 nM), were subjected to GEF assay as described above.

### Flow Cytometry analysis

CHO-S21 cells were plated at a density of 4 x 10^4^ cells per well on a 6-well tissue culture plate. Two days later the culture medium was replaced with 2 mL of fresh medium and cells were treated with indicated compounds for 24 hours. Immediately before analysis, the cells were washed with PBS and collected in PBS containing 4 mM EDTA. Single cell fluorescent signals (10,000 cells/sample) were measured by a dual-channel flow cytometry with LSRFortessa cell analyzer (Beckton Dickinson). GFP (excitation laser 488 nm, filter 530/30), Turquoise (modified CFP) (excitation laser 405 nm, filter 450/50) signals were detected. FlowJo software was used to analyze the data.

### Cell viability analysis (WST-1 assay)

CHO-S21 parental or VWM *c*ells were plated at a density of 2,000 cells per well in a 24-well tissue culture plate. Two days later the culture medium was replaced with the medium containing the indicated compounds as shown in Fig 4B. 24 hours later, the cells were washed once in regular medium and then maintained in regular medium for 48 hours. Then, the medium was replaced with fresh medium containing 50 μM WST-1 (Dojindo) and 20 μM 1-methoxy phenazine methosulfate (Sigma), and the cells were incubated for 2 hours in the cell culture incubator before absorbance at 440 nm in the culture media was measured.

### Statistical analysis

Statistical significances were determined by unpaired t test or one way ANOVA followed by Dunnett’s multiple comparisons test as indicated in the Figure legends, using Prism software (GraphPad Software, Inc.).

## Supporting Information

S1 Fig Supporting Figures related to Fig 1. (A) Time dependent decline of fluorescent intensity (FI) of eIF2-Bodipy-GDP substrate by purified wildtype (WT) or eIF2Bδ^A392D^ mutant (δA392D) eIF2B complex or control buffer (-). FI was measured every 20 seconds. (B) Coomassie brilliant blue stained SDS-PAGE of purified WT or δA392D eIF2B complexes used for measuring GEF activity in Fig 1B and S1A Fig. Note that the δA392D eIF2B complex possessed less eIF2Bε catalytic subunit when the same amounts of eIF2Bγ-FLAG were loaded (compare lane 2 and 3). The amount of purified eIF2B introduced into the GEF assay was adjusted to equalize the content of the eIF2Bε catalytic subunit. (C) GEF activity in the lysates of parental CHO-S21 and *EIF2B4*^*A392D*^ cells. Shown are means ± S.D. of three independent experiments. *P = 0.0066, Unpaired t test. (D) Quantitative PCR for mRNA expression of *EIF2B4* and *EIF2B1* in CHO-S21 parental and *EIF2B4*^*A392D*^ cells. Shown are means ± S.D. of three independent experiments. * P = 0.001, Unpaired t test. (E) Immunoblot of total eIF2Bδ and eIF2α in parental CHO-S21 and *EIF2B4*^*A392D*^ cell lysate. (F) Translation monitored by immunoblot for puromycinylated proteins in parental CHO-S21 and *EIF2B4*^*A392D*^ cells. Quantification of these measurements from four independent experiments is presented in Fig 4C. (G) Cell growth of parental CHO-S21 and *EIF2B4*^*A392D*^ cells under standard culture condition. Shown are the means ± S.D. of three independent experiments. Cells reached full confluence at day 5.
(PDF)

S2 Fig Supporting Figures related to Fig 2. (A) The *Cricetulus griseus EIF2B4* genomic locus as in Fig 1A, showing the position of the silent *Spe*I site introduced by recombination of the repair template encoding a wildtype protein and the one encoding the A392D mutation. Note that whilst the parental and repaired chromosomes could also be distinguished by loss of the *Pst*I site from the repaired version, a variable background of undigested PCR product eroded the discriminatory value of the *Pst*I RFLP. (B) Histogram of the *CHOP*::*GFP* reporter expression in untreated and histidinol-treated cells described in Fig 2C.
(PDF)

S3 Fig Supporting Figures related to Fig 3. (A) Allele structure of the *Cricetulus griseus EIF2B4* genomic locus (NW_003613640.1, 5027:5071 for R468W, 5084:5146 for R484W) targeted by CRISPR-Cas9 system and eIF2Bδ encoded protein (XP_003497100.1, 461:475 for R468W, 480:500 for R484W). Horizontal lines and vertical arrows represent the sgRNA binding sites and Cas9 cleavage sites, respectively. Mutations, shown in red, disrupt sgRNA targeting, eliminate or generate restriction enzyme site (a *Pst*I site for R468W or a *Sal*I site for R484W, respectively), and generate R468W or R484W mutation. The *EIF2B4*^R484W^ mutant clone possesses R484W mutation on one allele and 7 nucleotides deletion on another, which causes three missense mutations and subsequent premature stop codon. (B) Immunoblot of eIF2Bδ and eIF2α in parental and indicated VWM mutant CHO-S21 cells. (C) Schema of the experiment to measure the sensitivity for *EIF2S1*^*S51A*^ mutant cells to histidinol. After targeting the *EIF2S1* locus with an *EIF2S1*^*S51A*^ repair template, CHO-S21 cells were either left untreated (“Control”) or exposed to 0.5 mM histidinol for 2 days and allowed to recover for additional 2 days before treatment with 250 nM thapsigargin (Tg) for 1 day and flow cytometry to quantify the fraction of ISR negative (putative *EIF2S1*^*S51A*^ mutant) cells in the population. (D) Flow cytometry analysis of cells subjected to the experiment described in “C”. Note the depletion of *CHOP*::*GFP* negative, *XBP1*::*turquoise* positive putative *EIF2S1*^*S51A*^ mutant cells from the population of cells exposed to histidinol. (E) Flow cytometry analysis of reporter activity in parental CHO-S21 cells or a representative stable *EIF2S1*^*S51A*^ mutant clone (C8) isolated from the *EIF2S1*^*S51A*^ template-transfected CHO-S21 cell pool. Cells were treated with 2 μg/ml tunicamycin (Tm) for 20 hours before analysis. (F) Flow cytometry analysis of reporter activity in untreated (UT) and thapsigargin-treated (Tg) parental CHO-S7 or *EIF2B4*^*A392D*^ mutant cells following targeting of the *EIF2S1* locus with an *EIF2S1*^*S51A*^ repair template (as in Fig 3A). Note the lack of *CHOP*::*GFP* negative thapsigargin-treated putative *EIF2S1*^*S51A*^*; EIF2B4*^*A392D*^ double mutant cells (lower right panel) and the absence of a signal in the turquoise channel of these CHO-S7 cells lacking *XBP1*::*turquoise* reporter. (G) Percentage of *CHOP*::*GFP* negative thapsigargin-treated putative *EIF2S1*^*S51A*^ cells in the parental or *EIF2B4*^*A392D*^ population from experiments as in “F”. Shown are means ± S.D. of three independent experiments. ** P = 0.0021, Unpaired t test. (H) As in Fig 3A, but cells were transfected with an sgRNA/Cas9 plasmid targeting *ERN1*/IRE1α. Note the emergence of *CHOP*::*GFP* positive, *XBP1*::*turquoise* negative *ERN1*/IRE1α mutant cells in both the parental and *EIF2B4*^*A392D*^ pools. (I) Percentage of *CHOP*::*GFP* positive, *XBP1*::*turquoise* negative putative *ERN1*/IRE1α deleted cells in the parental or *EIF2B4*^*A392D*^ population from experiments shown in “H”. Shown are means ± S.D. of three independent experiments. No statistical significance in unpaired t test.
(PDF)

S1 Table Oligo DNA list. (PDF)
