# Supplementary figures and images for: Paradoxical Sensitivity to an Integrated Stress Response Blocking Mutation in Vanishing White Matter Cells
Source: PLoS One. 2016 Nov 3;11(11):e0166278. doi: 10.1371/journal.pone.0166278 (PMC5094784; doi:10.1371/journal.pone.0166278)

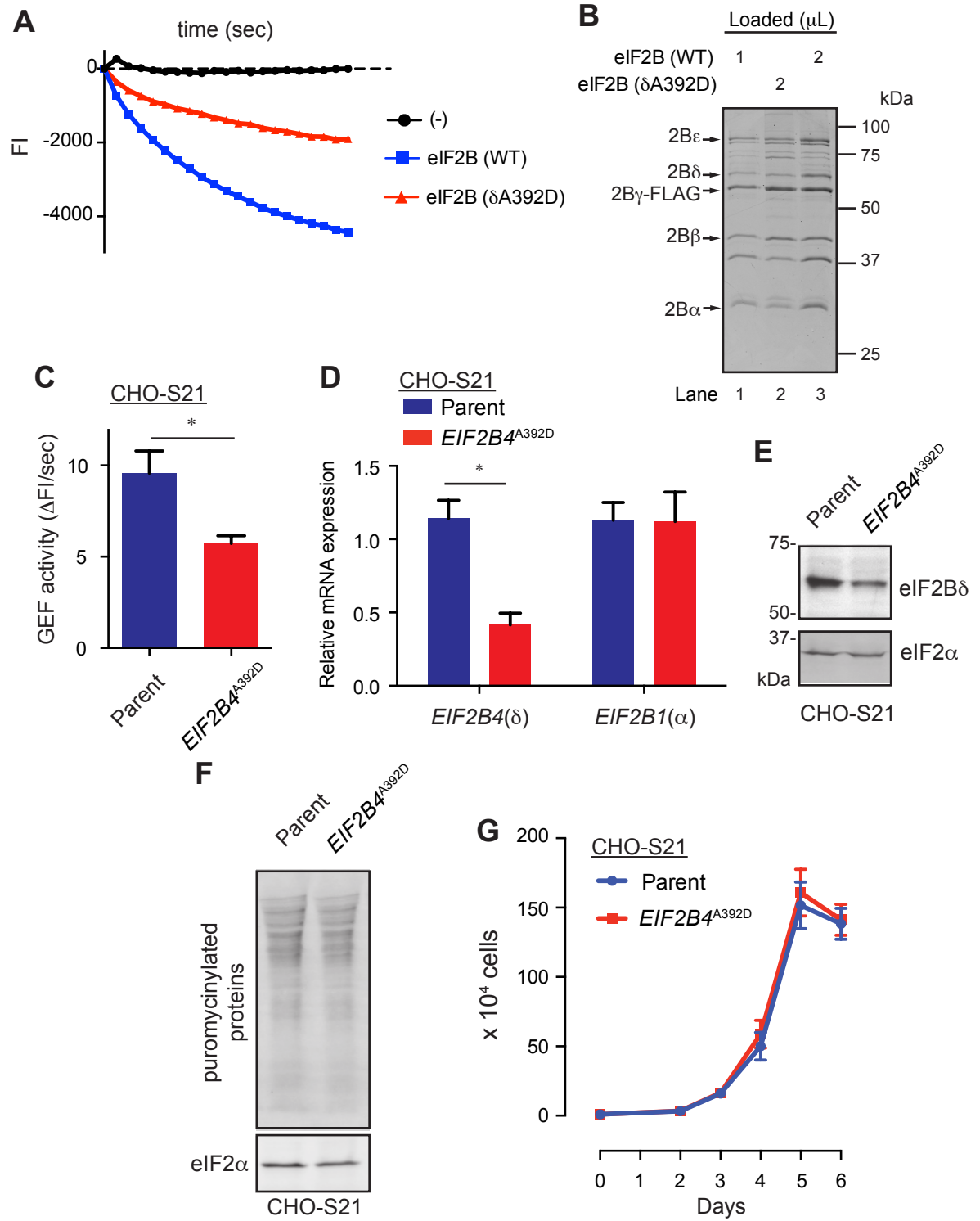

Supplement: S1 Fig — (A) Time dependent decline of fluorescent intensity (FI) of eIF2-Bodipy-GDP substrate by purified wildtype (WT) or eIF2BδA392D mutant (δA392D) eIF2B complex or control buffer (-). FI was measured every 20 seconds. (B) Coomassie brilliant blue stained SDS-PAGE of purified WT or δA392D eIF2B complexes used for measuring GEF activity in Fig 1B and S1A Fig. Note that the δA392D eIF2B complex possessed less eIF2Bε catalytic subunit when the same amounts of eIF2Bγ-FLAG were loaded (compare lane 2 and 3). The amount of purified eIF2B introduced into the GEF assay was adjusted to equalize the content of the eIF2Bε catalytic subunit. (C) GEF activity in the lysates of parental CHO-S21 and EIF2B4A392D cells. Shown are means ± S.D. of three independent experiments. *P = 0.0066, Unpaired t test. (D) Quantitative PCR for mRNA expression of EIF2B4 and EIF2B1 in CHO-S21 parental and EIF2B4A392D cells. Shown are means ± S.D. of three independent experiments. * P = 0.001, Unpaired t test. (E) Immunoblot of total eIF2Bδ and eIF2α in parental CHO-S21 and EIF2B4A392D cell lysate. (F) Translation monitored by immunoblot for puromycinylated proteins in parental CHO-S21 and EIF2B4A392D cells. Quantification of these measurements from four independent experiments is presented in Fig 4C. (G) Cell growth of parental CHO-S21 and EIF2B4A392D cells under standard culture condition. Shown are the means ± S.D. of three independent experiments. Cells reached full confluence at day 5. (PDF) [file pone.0166278.s001.pdf]

**A**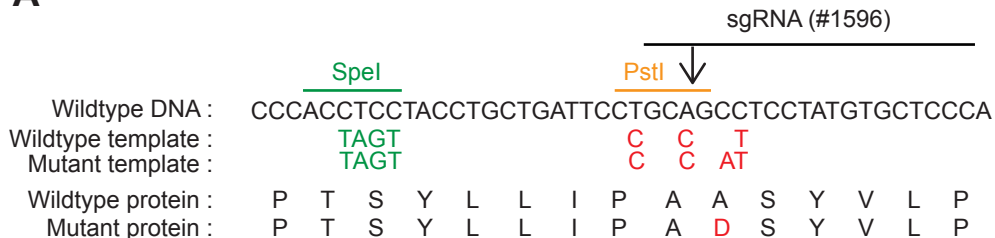**B**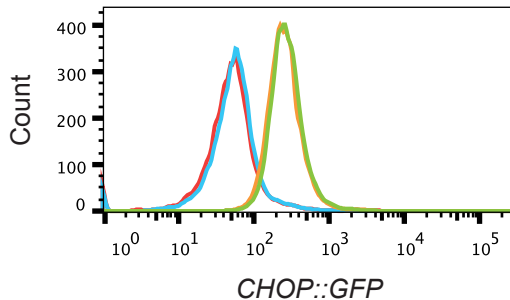

- WT temp pool (UT)
- A392D temp pool (UT)
- WT temp pool (His)
- A392D temp pool (His)

Supplement: S2 Fig — (A) The Cricetulus griseus EIF2B4 genomic locus as in Fig 1A, showing the position of the silent SpeI site introduced by recombination of the repair template encoding a wildtype protein and the one encoding the A392D mutation. Note that whilst the parental and repaired chromosomes could also be distinguished by loss of the PstI site from the repaired version, a variable background of undigested PCR product eroded the discriminatory value of the PstI RFLP. (B) Histogram of the CHOP::GFP reporter expression in untreated and histidinol-treated cells described in Fig 2C. (PDF) [file pone.0166278.s002.pdf]

**A*****R468W***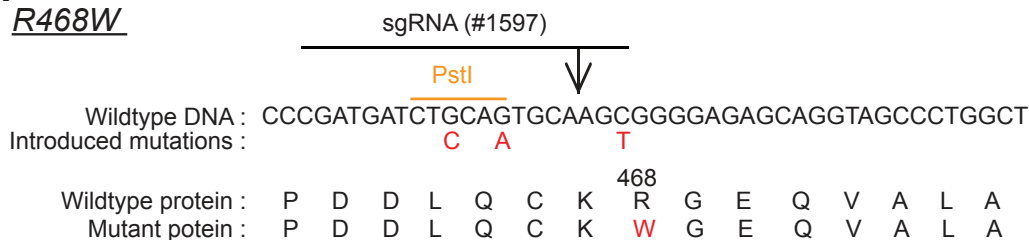***R484W***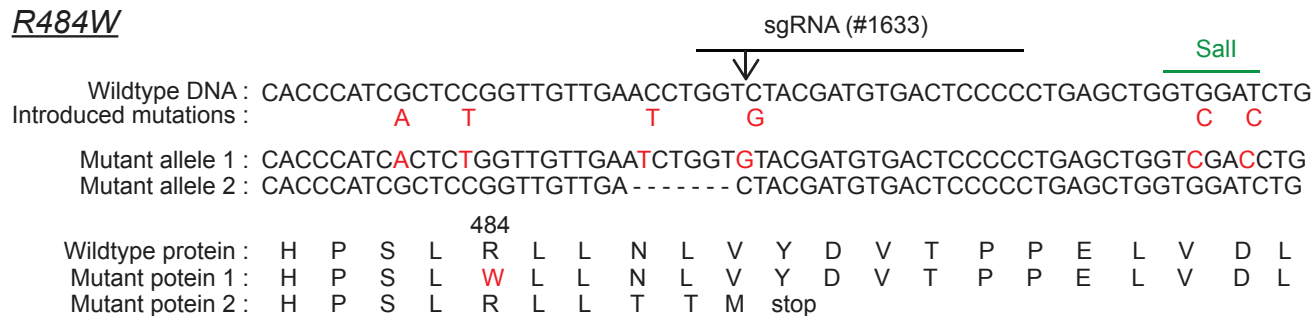**B**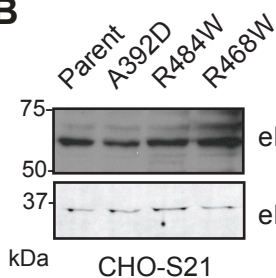**C**

Fig 3B S51A template-transfected cells

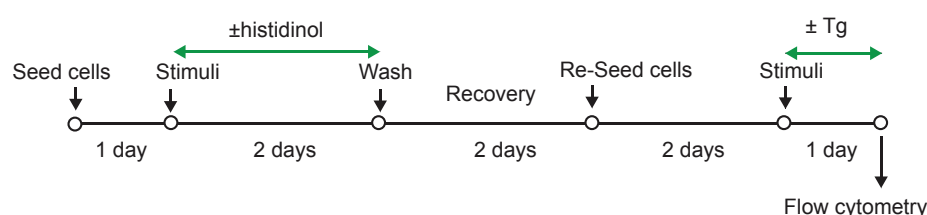**D**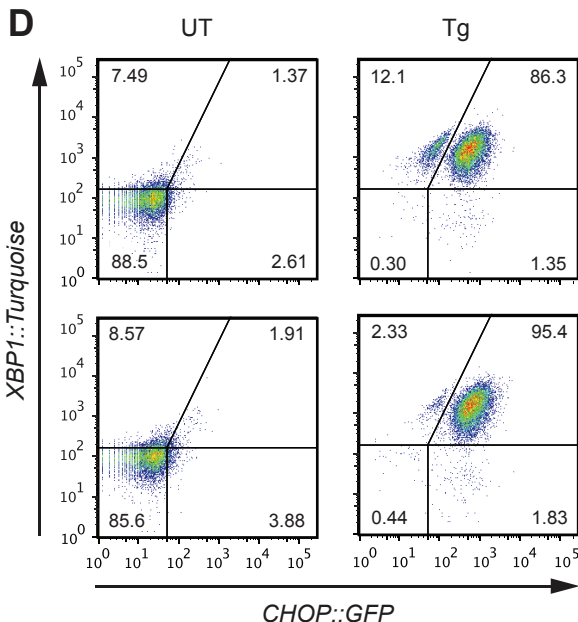**E**

CHO-S21

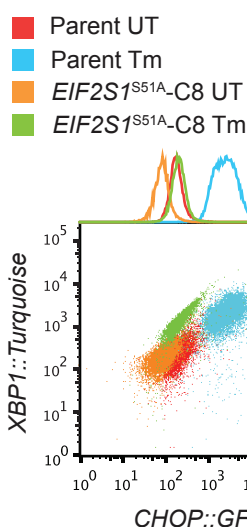**F**

CHO-S7

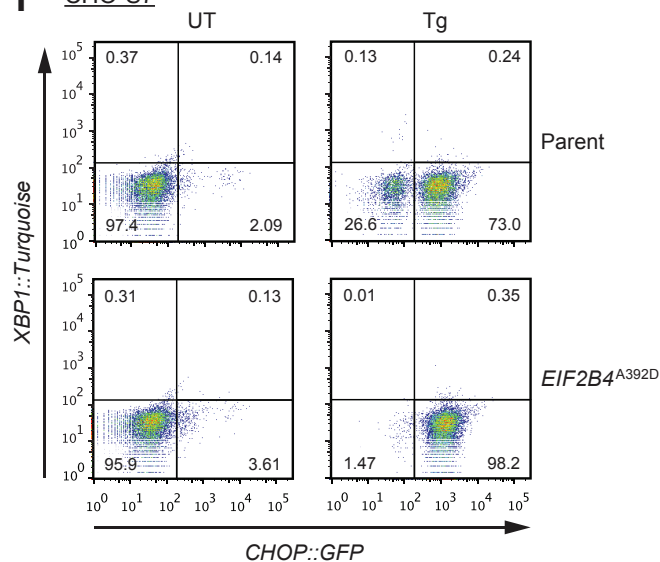**H**

CHO-S21

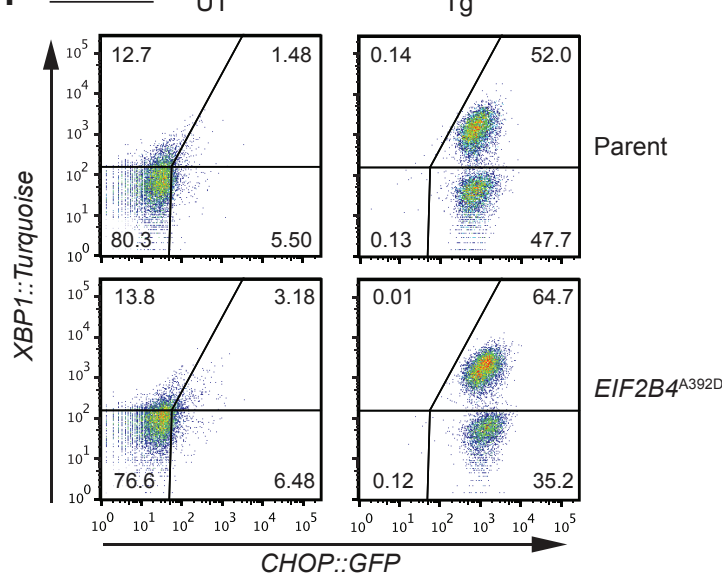**G**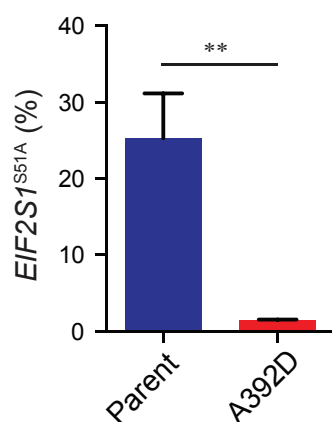**I**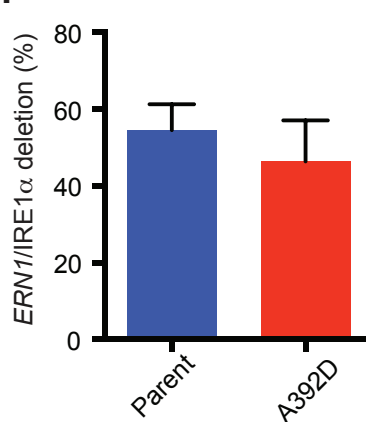

Supplement: S3 Fig — (A) Allele structure of the Cricetulus griseus EIF2B4 genomic locus (NW_003613640.1, 5027:5071 for R468W, 5084:5146 for R484W) targeted by CRISPR-Cas9 system and eIF2Bδ encoded protein (XP_003497100.1, 461:475 for R468W, 480:500 for R484W). Horizontal lines and vertical arrows represent the sgRNA binding sites and Cas9 cleavage sites, respectively. Mutations, shown in red, disrupt sgRNA targeting, eliminate or generate restriction enzyme site (a PstI site for R468W or a SalI site for R484W, respectively), and generate R468W or R484W mutation. The EIF2B4R484W mutant clone possesses R484W mutation on one allele and 7 nucleotides deletion on another, which causes three missense mutations and subsequent premature stop codon. (B) Immunoblot of eIF2Bδ and eIF2α in parental and indicated VWM mutant CHO-S21 cells. (C) Schema of the experiment to measure the sensitivity for EIF2S1S51A mutant cells to histidinol. After targeting the EIF2S1 locus with an EIF2S1S51A repair template, CHO-S21 cells were either left untreated (“Control”) or exposed to 0.5 mM histidinol for 2 days and allowed to recover for additional 2 days before treatment with 250 nM thapsigargin (Tg) for 1 day and flow cytometry to quantify the fraction of ISR negative (putative EIF2S1S51A mutant) cells in the population. (D) Flow cytometry analysis of cells subjected to the experiment described in “C”. Note the depletion of CHOP::GFP negative, XBP1::turquoise positive putative EIF2S1S51A mutant cells from the population of cells exposed to histidinol. (E) Flow cytometry analysis of reporter activity in parental CHO-S21 cells or a representative stable EIF2S1S51A mutant clone (C8) isolated from the EIF2S1S51A template-transfected CHO-S21 cell pool. Cells were treated with 2 μg/ml tunicamycin (Tm) for 20 hours before analysis. (F) Flow cytometry analysis of reporter activity in untreated (UT) and thapsigargin-treated (Tg) parental CHO-S7 or EIF2B4A392D mutant cells following targeting of the EIF2S1 locus with an [file pone.0166278.s003.pdf]
