# Supplementary material for: Paradoxical Sensitivity to an Integrated Stress Response Blocking Mutation in Vanishing White Matter Cells
Source: PLoS One. 2016 Nov 3;11(11):e0166278. doi: 10.1371/journal.pone.0166278 (PMC5094784; doi:10.1371/journal.pone.0166278)
Supplement: S1 Table — (PDF) [file pone.0166278.s004.pdf]

S1 Table Oligo DNA list

No.	Name	Oligo Sequence	Used for
1	haEIF2B3_g299541F_981	CACCGGCGAATGAACGAGGTAATTG	Construction for an sgRNA/Cas9 plasmid targeting EIF2B3
2	haEIF2B3_g299541R_982	AAACCAATTACCTCGTTCATTCGCC	Construction for an sgRNA/Cas9 plasmid targeting EIF2B3
3	haEIF2B3_Int_S_SacI_977	GAAGAGCTCCAAAGCATCCATGCTAT	Construction for an EIF2B3_3XFLAG repair template
4	haEIF2B3_Ex11_AS_EcoRI_978	CAGGAATTCGATCTCCATGAGTTGATCACTTCCTACAATTACCTCGTTCAT	Construction for an EIF2B3_3XFLAG repair template
5	haEIF2B3_Ex11_S_ApaI_979	TGAGGGCCCGTCCTGAACAAGCCTGACCCCT	Construction for an EIF2B3_3XFLAG repair template
6	haEIF2B3_3genome_AS_KpnI_980	ACCGGTACCTTTAATTCTTGATCCTCCTGC	Construction for an EIF2B3_3XFLAG repair template
7	haEIF2B3_5OUT_S_1029	TGCATGCATGTGGTATACGTACGTG	Genotyping for EIF2B3_3FLAG insertion
8	haEIF2B3_OUT_AS _992	CAGCCCTTTAATTCTTGATCCTCCT	Genotyping for EIF2B3_3FLAG insertion
9	haEIF2B4_g257792F_1171	CACCGGGCTGCAGGAATCAGCAGGT	Construction for an sgRNA/Cas9 plasmid targeting EIF2B4
10	haEIF2B4_g257792R_1172	AAACACCTGCTGATTCCTGCAGCCC	Construction for an sgRNA/Cas9 plasmid targeting EIF2B4
11	haEIF2B4_g257788F_1173	CACCGGGGAGCACATAGGAGGCTGC	Construction for an sgRNA/Cas9 plasmid targeting EIF2B4
12	haEIF2B4_g257788R_1174	AAACGCAGCCTCCTATGTGCTCCCC	Construction for an sgRNA/Cas9 plasmid targeting EIF2B4
13	haEIF2B4_EX10 _1F_1161	CTTAGAGAAGCCATTGATCGG	Construction for an EIF2B4 A392D repair template
14	haEIF2B4_A392D_1R_1162	AGCACATAGGAATCGGCGGGAATCAGCAGGTAGGAGGTGG	Construction for an EIF2B4 A392D repair template
15	haEIF2B4_A392D_1F_1163	CCCGCCGATTCCTATGTGCTCCCAGAGGTGA	Construction for an EIF2B4 A392D repair template
16	haEIF2B4_INT11_1R_1164	TAAGGGCCAGTGAGGTGGCTCAAT	Construction for an EIF2B4 A392D repair template
17	haEIF2B4_INT9_OUT1F_1159	CTCAACATTTAGGCAAAGTCAG	Genotyping for EIF2B4 A392 locus
18	haEIF2B4_INT11_OUT1R_1160	TATAGGCTACACAGAGAAACCC	Genotyping for EIF2B4 A392 locus
19	haEIF2B4_qPCR_L1_1496	CCGGTGTATCACACTGCTTC	qPCR primer
20	haEIF2B4_qPCR_R1_1497	CTTCATTGGGAGGTGTTGTG	qPCR primer
21	haEIF2B1_qPCR_L1_1498	GATGCTGCTGTTGGCTACAT	qPCR primer
22	haEIF2B1_qPCR_R1_1499	ATTCCTCCGTTCTCAACCAC	qPCR primer
23	haRpl27_1S_1489	ACAATCACCTCATGCCCACAAG	qPCR primer
24	haRpl27_1AS_1490	GCGTTTCAGGGCTGGGTCTC	qPCR primer
25	CHO_eif2s1_CrispyC_1s_1017	CACCGGATCAGTTTGTTTATAGAA	Construction for an sgRNA/Cas9 plasmid targeting eIF2a
26	CHO_eif2s1_CrispyC_2AS_1020	AAACTTCTATAAACAAACTGATCC	Construction for an sgRNA/Cas9 plasmid targeting eIF2a
27	CHO_IRE1_g_TM_s7_s_1151	CACCGCCCCGTTGACTCCATGCTCA	Construction for an sgRNA/Cas9 plasmid targeting IRE1a
28	CHO_IRE1_g_TM_s7_as_1152	AAACTGAGCATGGAGTCAACGGGGC	Construction for an sgRNA/Cas9 plasmid targeting IRE1a
29	CHO_eif2s1_ssODN_C_1022	AATGTAAGATCCATAGCTGAGATGGGAGCCTATGTTAGCTTGTTGGAATAC<br>AATAACATTGAAGGCATGATTCTTCTTAGTGAATTA**G**CCAGAAGACGTAT**T**<br>CGTTCTATAAACAAACTGATCCGCATTGGCAGAAATGAATGTGTAGTTGTC<br>ATTAGAGTGGACAAAGAAAAAGGTAAGTGAGAAAAA	eIF2α S51A repair template
30	CHO_eif2s1_S51_WT_SacI_ssODN_1411	AATGTAAGATCCATAGCTGAGATGGGAGCCTATGTTAGCTTGTTGGAATAC<br>AATAACATTGAAGGCATGATTCTTCTTAGTGAGCTCTCCAGAAGACGTATT<br>CGTTCTATAAACAAACTGATCCGCATTGGCAGAAATGAATGTGTAGTTGTC<br>ATTAGAGTGGACAAAGAAAAAGGTAAGTGAGAAAAA	eIF2α S51 WT repair template
31	haEIF2B4_A392D_v2_1R_1302	ATAGGAATCGGCGGGAATCAGCAGGTAACTAGTGGGGACCCCAGCAT	Construction for an EIF2B4 A392D repair template
32	haEIF2B4_A392WT_1R_1303	ATAGGAAGCGGCGGGAATCAGCAGGTAACTAGTGGGGACCCCAGCAT	Construction for an EIF2B4 A392 WT repair template
33	haEIF2B4_g257760F_1254	CACCGGGGGAGTCACATCGTAGACC	Construction for an sgRNA/Cas9 plasmid targeting EIF2B4
34	haEIF2B4_g257760R_1255	AAACGGTCTACGATGTGACTCCCCC	Construction for an sgRNA/Cas9 plasmid targeting EIF2B4
35	haEIF2B4_EX12_1F_1167	TCATGCACTCCTGGCCAATGGAT	Construction for an EIF2B4 R484W repair template
36	haEIF2B4_R484W_v2_1R_1250	AGTCACATCGTACACCAGATTCAACAACCAGAGTGATGGGTGGTTCTGCCAG<br>TT	Construction for an EIF2B4 R484W repair template
37	haEIF2B4_R484W_v2_1F_1251	TCTGGTGTACGATGTGACTCCCCCTGAGCTGGTCGACCTGGTGATCACAGAA	Construction for an EIF2B4 R484W repair template
38	haEIF2B4_3UTR_1R_1170	CATTATGTAGACTAGGCTGGCC	Construction for an EIF2B4 R484W repair template
39	haEIF2B4_g257776F_1175	CACCGGGGGAGTCACATCGTAGACC	Construction for an sgRNA/Cas9 plasmid targeting EIF2B4
40	haEIF2B4_g257776R_1176	CACCGGCTTGCACTGCAGATCATCG	Construction for an sgRNA/Cas9 plasmid targeting EIF2B4
41	haEIF2B4_ R484W_1R_1168	TCTCCCCACTTGCATTGGAGATCATCGGGGTCATCTGCAA	Construction for an EIF2B4 R468W repair template
42	haEIF2B4_R484W_1F_1169	CTCCAATGCAAGTGGGGAGAGCAGGTAGCCCTGGCTAA	Construction for an EIF2B4 R468W repair template
